# Gene Signature of High White Blood Cell Count in B-Precursor Acute Lymphoblastic Leukemia

**DOI:** 10.1371/journal.pone.0161539

**Published:** 2016-08-18

**Authors:** Holly Edwards, Mara Rubenstein, Alan A. Dombkowski, J. Timothy Caldwell, Roland Chu, Ana C. Xavier, Ryan Thummel, Melody Neely, Larry H. Matherly, Yubin Ge, Jeffrey W. Taub

**Affiliations:** 1 Department of Oncology, Wayne State University School of Medicine, Detroit, MI, United States of America; 2 Molecular Therapeutics Program, Barbara Ann Karmanos Cancer Institute, Wayne State University School of Medicine, Detroit, MI, United States of America; 3 Division of Pediatric Hematology/Oncology, Children’s Hospital of Michigan, Detroit, MI, United States of America; 4 Division of Clinical Pharmacology and Toxicology, Children’s Hospital of Michigan, Detroit, Michigan, United States of America; 5 Department of Pediatrics, Wayne State University School of Medicine, Detroit, MI, United States of America; 6 MD/PhD Program, Wayne State University School of Medicine, Detroit, MI, United States of America; 7 Cancer Biology Program, Wayne State University School of Medicine, Detroit, MI, United States of America; 8 Division of Pediatric Hematology/Oncology, Children’s Hospital of Alabama, University of Alabama at Birmingham, Birmingham, AL, United States of America; 9 Department of Anatomy and Cell Biology, Wayne State University School of Medicine, Detroit, MI, United States of America; 10 Department of Immunology and Microbiology, Wayne State University School of Medicine, Detroit, MI, United States of America; 11 Department of Pharmacology, Wayne State University School of Medicine, Detroit, MI, United States of America; German Cancer Research Center (DKFZ), GERMANY

## Abstract

In this study we sought to identify genetic factors associated with the presenting white blood cell (WBC) count in B-precursor acute lymphoblastic leukemia (BP-ALL). Using *ETV6-RUNX1*-positive BP-ALL patient samples, a homogeneous subtype, we identified 16 differentially expressed genes based on the presenting WBC count (< 50,000/cumm vs > 50,000). We further confirmed that *IL1R1*, *BCAR3*, *KCNH2*, *PIR*, and *ZDHHC23* were differentially expressed in a larger cohort of *ETV6-RUNX1*-negative BP-ALL patient samples. Statistical analysis demonstrated that expression levels of these genes could accurately categorize high and low WBC count subjects using two independent patient sets, representing positive and negative *ETV6-RUNX1* cases. Further studies in leukemia cell line models will better delineate the role of these genes in regulating the white blood cell count and potentially identify new therapeutic targets.

## Introduction

**Fig 1:**
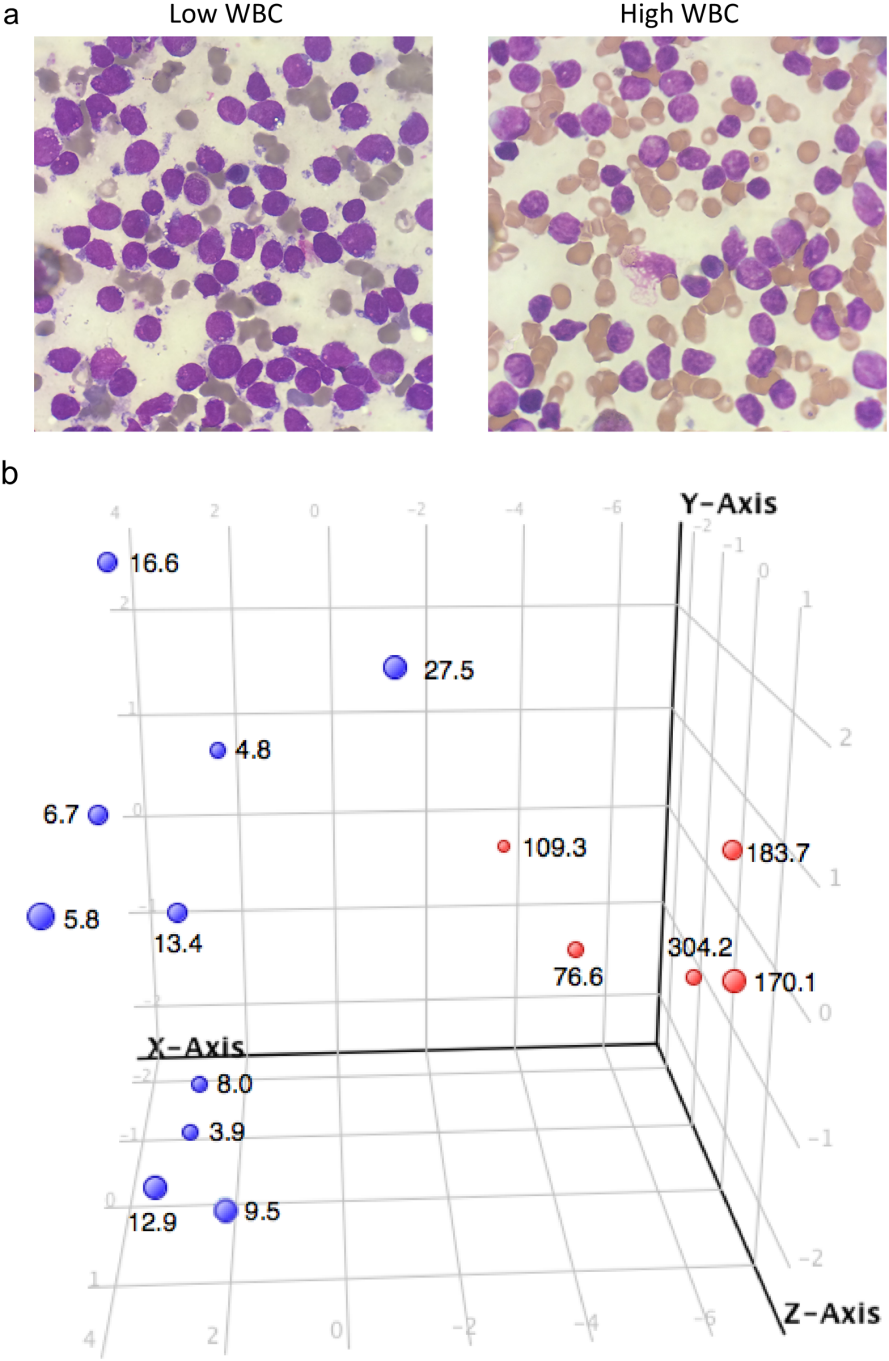
Differentially expressed features between high and low WBC count *ETV6-RUNX1*-positive patient samples. (**a**) Diagnostic bone marrow aspiration samples from a patient with low (left side) and high (right side) WBC count at initial presentation were Wright-Giesma stained. (**b**) Total RNAs were isolated from 15 *ETV6-RUNX1*-positive patient samples. Aminyoallyl-aRNA was produced using 100 ng total RNA. Gene expression microarray was performed using the Agilent 60-mer oligo array (Human Gene Expression V2, 8X60K). Slides were scanned with the Agilent dual laser scanner with SureScan High Resolution Technology. Tiff images were analyzed using Agilent’s feature extraction software version 11.0.1.1 to obtain fluorescent intensities for each spot on the array. Probes that were not detected significantly above background in at least 75% of the samples for at least any one category (low or high WBC) were filtered out, leaving 28,973 probes. Differentially expressed genes were identified by *P*-values ≤ 0.05, which were calculated using a moderated t-test with Westfall and Young multiple test corrections. Principal component analysis using the 26 differentially expressed features was performed. Data points are labeled with WBC count (x 10^3^/cumm).

Acute lymphoblastic leukemia (ALL) is the most common pediatric cancer. Over the last 30 years, the 5-year survival rate has significantly improved, increasing from 57% to 90% [1]. The introduction of risk-stratified therapy has contributed significantly to the improved survival [2]. For B-precursor ALL (BP-ALL), the National Cancer Institute has defined two risk groups based on age and presenting white blood cell (WBC) count, which continue to remain important prognostic factors today [2]. The standard risk group consists of patients with a presenting WBC count < 50,000/cumm and between 1 and 10 years of age, whereas the high risk group has a WBC count ≥ 50,000/cumm and/or age ≥ 10 years [3]. To date, factors regulating the presenting WBC count and its association with prognosis in BP-ALL remain unknown. At diagnosis, there is essentially complete replacement of normal hematopoiesis with blast cells and there are no differentiating morphologic features between patients with low and high WBC count (Fig 1a). Based on this, it is hard to envision why, for example, a one log difference in peripheral WBC (e.g. 10,000/cumm vs 100,000/cumm) can have such a major impact on prognosis. Thus, identifying genetic factors associated with the regulation of WBCs in the peripheral circulation may improve our understanding of this disease and potentially lead to the development of new therapies.

BP-ALL is complex and heterogeneous which could complicate the identification of genetic factors linked to the peripheral WBC count. Therefore, we selected the largest molecular subgroup, *ETV6-RUNX1*-positive BP-ALL accounting for ~25% of pediatric ALL cases, to identify genes linked to the peripheral WBC count. Although these cases are usually associated with low-risk features, up to 25% of cases have a presenting WBC count > 50,000/cumm. We found that *IL1R1* (Il1r1 interleukin 1 receptor, type I), *BCAR3* (breast cancer anti-estrogen resistance 3), *KCNH2* (potassium channel, voltage gated related subfamily H, member 2), *PIR* (pirin), and *ZDHHC23* (zinc finger, DHHC-type containing 23) were differentially expressed. These findings were further confirmed in a larger cohort of samples from patients with *ETV6-RUNX1*-negative BP-ALL, thus identifying a unique gene expression signature for high WBC in BP-ALL.

## Methods

### Clinical Samples

Diagnostic bone marrow samples with *de novo* ALL were obtained from the Children’s Hospital of Michigan leukemia cell bank. Mononuclear cells were purified by standard Ficoll-Hypaque density centrifugation. Written informed consent was provided by the parent or legal guardian according to the Declaration of Helsinki. Sample handling and data analysis protocols were approved by the Human Investigation Committee of the Wayne State University School of Medicine.

### Gene Expression Microarray Analysis

Total RNAs were extracted using TRIzol according to the manufacturer’s instructions (Life Technologies, Carlsbad, CA). Aminoallyl-aRNA was produced using TargetAMP 1-Round Aminoallyl-aRNA Amplification Kit 101 (Epicentre, Madison, WI) and Agilent Spike-in Controls for one color microarrays according to the manufacturer’s protocol (Agilent Technologies, Santa Clara, CA). Three μg of each aminoallyl-aRNA sample was incubated with Alexa Fluor 555 (Life Technologies) for 30 min at room temperature and then run through the RNeasy Mini Elute column (Qiagen, Valencia, CA) to remove unincorporated dye. The samples were prepared for hybridization following the Agilent “One-Color Microarray-Based GE Analysis” protocol. Six hundred ng of Alexa Fluor 555 labeled aminoallyl-aRNA was used to hybridize to the Agilent 60-mer oligo array (Human Gene Expression V2, 8X60K). The data has been deposited in the GEO repository, accession number GSE63370.

### Quantification of Gene Expression by Real-time RT-PCR

cDNAs were prepared, as previously described [4]. Transcripts were quantitated using TaqMan probes (Life Technologies) and a LightCycler LC480 real-time PCR machine (Roche Diagnostics, Indianapolis, IN), based on the manufacturer’s instructions.

### Protein Interaction Network Analysis

Protein interaction analysis was performed using STRING (Search Tool for the Retrieval of Interacting Genes/Proteins, string-db.org) (PMID 25352553). Data settings in the program were: active interaction sources = all; minimum required interaction score = 0.150; max number of interactors, first shell = 20, second shell = 10. Gene ontology (GO) analysis was performed within STRING using the GO biological process.

## Results and Discussion

**Fig 2:**
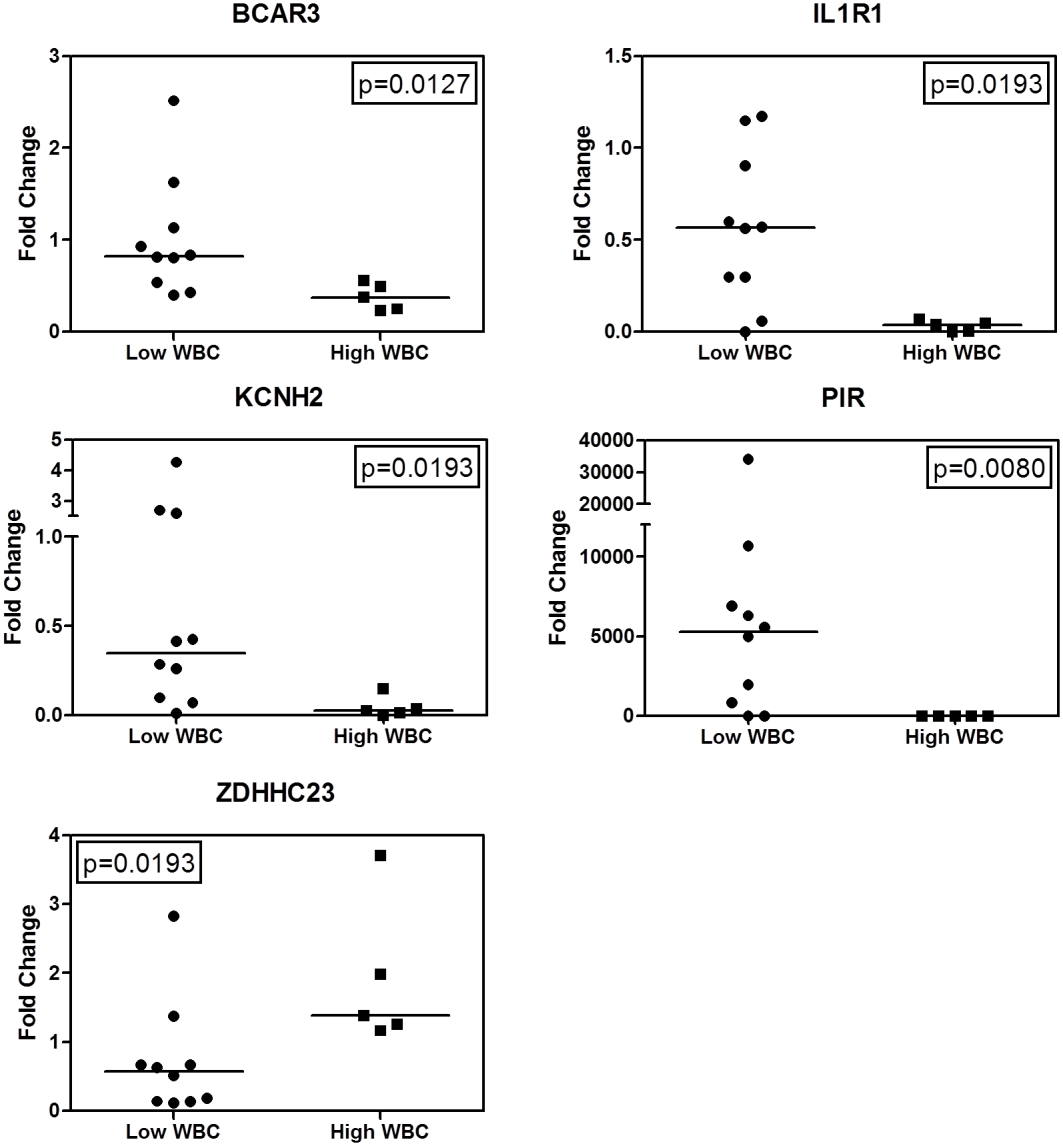
*BCAR3*, *IL1R1*, *KCNH2*, *PIR*, and *ZDHHC23* expression levels were significantly different between low and high WBC *ETV6-RUNX1*-positive patient samples. Total RNAs from *ETV6-RUNX1*-positive patient samples were isolated. cDNA was prepared using 1 μg total RNA and transcript levels were determined by real-time RT-PCR. Transcript levels were normalized to *RPL13a* and relative expression levels were calculated using the comparative Ct method (comparing all samples to the expression levels in the *ETV6-RUNX1*-positive REH cell line). The horizontal lines indicate the median. The *P*-values were calculated using the Mann-Whitney *U*-test and GraphPad Prism 5.0 software.

**Fig 3:**
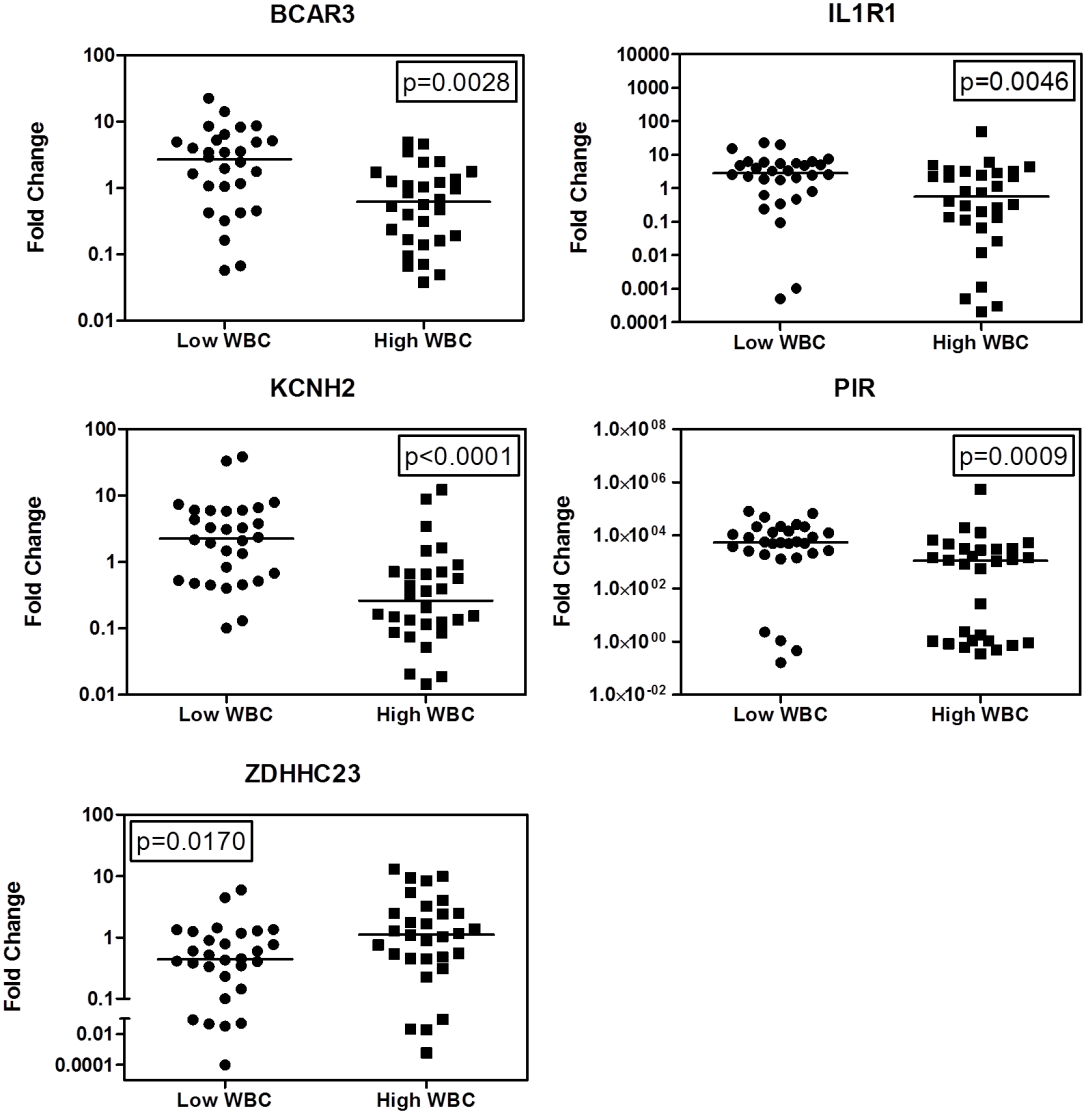
Expression of *BCAR3*, *IL1R1*, *KCNH2*, *PIR*, and *ZDHHC23* were significantly different between the low and high WBC *ETV6-RUNX1*-negative patient samples. Total RNAs from *ETV6-RUNX1*-negative patient samples were isolated. cDNA was prepared using 1 μg total RNA and transcript levels were determined by real-time RT-PCR. Transcript levels were normalized to *RPL13a* and relative expression levels were calculated using the comparative Ct method (comparing all samples to the expression levels in the *ETV6-RUNX1*-positive REH cell line). The horizontal lines indicate the median. The *P*-values were calculated using the Mann-Whitney *U*-test and GraphPad Prism 5.0 software.

In this study, we examined the gene expression profile of 15 diagnostic *ETV6-RUNX1*-positive primary patient samples (age < 10 years) by gene expression microarray, comparing patients with low vs high WBC count (Table 1). We found 26 differentially expressed features (probes, *P*-values ≤ 0.05). Principal component analysis revealed sample clustering based on the combined profiles of these features, with high WBC samples clustering on the right and low WBC on the left (Fig 1b). These results demonstrated that the samples could be separated by the expression profiles of the 26 differentially expressed features. The probe IDs were then cross-referenced with Entrez Gene IDs, which provided 16 gene IDs. Validation of the microarray results were performed using real-time RT-PCR. When comparing low and high WBC samples, *BCAR3* (*P* = 0.0127), *IL1R1* (*P* = 0.0193), *KCNH2* (*P* = 0.0193), *PIR* (*P* = 0.0080), *ZDHHC23* (*P* = 0.0193), *SLC5A11* (*P* = 0.0047), *JAKMIP1* (*P* = 0.0047), and *P2RX7* (*P* = 0.0047) expression levels were significantly different (Fig 2). To extend our findings, we measured the expression levels of these genes in 60 randomly selected *ETV6-RUNX1*-negative pediatric BP-ALL samples (Table 2). Expression of *BCAR3* (*P* = 0.0028), *IL1R1* (*P* = 0.0046), *KCNH2* (*P* < 0.0001), *PIR* (*P* = 0.0009), and *ZDHHC23* (*P* = 0.0170) were significantly different between the low and high WBC samples (Fig 3). Then we used logistic regression to create a predictive model from these five genes measured by RT-PCR in the 60 *ETV6-RUNX*1-negative pediatric BP-ALL cases and obtained a predictive model with a goodness of fit *P*-value < 0.0001. Furthermore, the predictive power of the model was evident from receiver operating characteristic (ROC) curve analysis. The area under the curve was 0.88 (data not shown), indicating a very good predictive accuracy in classifying samples as either high or low WBC based only on the expression pattern of the five genes.

**Table 1:** *ETV6-RUNX1*-positive patient characteristics.

Age (years)	Sex	Race	WBC (x10^3^/cumm)	Hgb (gm/dL)	Platelet count (x10^3^/cumm)	Sample type
2	F	W	9.5	3.6	14	BM
2	M	W	16.6	10.7	176	BM
3	M	B	3.9	9	217	BM
3	M	W	4.8	5.6	42	BM
3	M	W	27.5	4.9	7	PB
4	M	W	13.4	9.4	112	BM
5	M	W	5.8	7	71	BM
5	M	W	6.7	8.5	286	BM
5	F	B	12.9	8.3	32	BM
8	F	B	8	8.6	214	BM
2	M	W	109.3	6.4	66	BM
3	F	W	76.6	9	121	PB
3	F	W	170.1	5.4	24	PB
4	M	W	183.7	7.2	28	BM
6	F	B	304.2	3	31	BM

F, female; M, male; W, Caucasian; B, African American; BM, bone marrow; PB, peripheral blood; WBC, white blood cell.

**Table 2:** *ETV6-RUNX1*-negative patient characteristics.

Sample ID	race	nci	bcr	e2a	tel	hyperdiploid	Age group	WBC group
B10211	White	SR	No	No	No	Yes	<10 yrs	<50
B10224	White	SR	No	No	No	No	<10 yrs	<50
B10226	White	SR	No	No	No	Yes	<10 yrs	<50
B10228	White	SR	No	No	No	No	<10 yrs	<50
B10231	White	SR	No	No	No	Yes	<10 yrs	<50
B10233	White	SR	No	No	No	Yes	<10 yrs	<50
B10235	Other	HR	No	No	No	No	10+ yrs	<50
B10237	White	SR	No	No	No	Yes	<10 yrs	<50
B10250	Black	SR	No	No	No	Yes	<10 yrs	<50
B10253	White	SR	No	No	No	Yes	<10 yrs	<50
B10254	Hispanic	SR	No	No	No	Yes	<10 yrs	<50
B10255	White	HR	No	No	No	No	10+ yrs	<50
B10256	Other	SR	No	No	No	Yes	<10 yrs	<50
B10257	Black	HR	No	No	No	No	10+ yrs	<50
B10262	White	SR	No	No	No	No	<10 yrs	<50
B10267	Hispanic	HR	No	No	No	No	10+ yrs	<50
B10272	Hispanic	HR	No	No	No	No	10+ yrs	<50
B10275	White	SR	No	No	No	No	<10 yrs	<50
B10279	White	HR	No	No	No	No	10+ yrs	<50
B10286	White	SR	No	No	No	No	<10 yrs	<50
B10288	White	SR	No	No	No	No	<10 yrs	<50
B10289	White	SR	No	No	No	Yes	<10 yrs	<50
B10290	Hispanic	SR	No	No	No	Yes	<10 yrs	<50
B10291	Hispanic	HR	No	Yes	No	No	10+ yrs	<50
B10294	Other	SR	No	No	No	Yes	<10 yrs	<50
B10296	Hispanic	HR	No	No	No	No	10+ yrs	<50
B10297	White	SR	No	No	No	No	<10 yrs	<50
B10300	White	SR	No	No	No	Yes	<10 yrs	<50
B10301	White	HR	No	No	No	No	10+ yrs	<50
B10306	Other	SR	No	No	No	No	<10 yrs	<50
B10212	White	HR	No	No	No	No	<10 yrs	50+
B10214	White	HR	No	No	No	No	<10 yrs	50+
B10217	White	HR	No	No	No	No	10+ yrs	50+
B10223	White	HR	No	No	No	No	<10 yrs	50+
B10227	White	HR	No	No	No	Yes	<10 yrs	50+
B10229	White	HR	No	No	No	No	<10 yrs	50+
B10232	Hispanic	HR	No	No	No	No	<10 yrs	50+
B10234	White	HR	No	No	No	No	<10 yrs	50+
B10236	White	HR	No	Yes	No	No	<10 yrs	50+
B10242	Black	HR	No	Yes	No	No	10+ yrs	50+
B10243	White	HR	No	No	No	No	<10 yrs	50+
B10245	Other	HR	Yes	No	No	No	<10 yrs	50+
B10247	White	HR	No	No	No	No	10+ yrs	50+
B10249	White	HR	No	No	No	No	<10 yrs	50+
B10258	White	HR	No	No	No	No	10+ yrs	50+
B10260	White	HR	No	No	No	No	<10 yrs	50+
B10265	Hispanic	HR	No	Yes	No	No	<10 yrs	50+
B10266	White	HR	No	No	No	No	<10 yrs	50+
B10268	White	HR	No	No	No	No	<10 yrs	50+
B10270	White	HR	No	No	No	No	<10 yrs	50+
B10276	White	HR	No	No	No	No	<10 yrs	50+
B10282	White	HR	No	No	No	No	<10 yrs	50+
B10283	Hispanic	HR	No	No	No	No	<10 yrs	50+
B10285	White	HR	No	No	No	No	<10 yrs	50+
B10292	White	HR	No	No	No	No	<10 yrs	50+
B10293	White	HR	No	Yes	No	No	<10 yrs	50+
B10295	White	HR	No	No	No	Yes	<10 yrs	50+
B10299	White	HR	No	No	No	No	<10 yrs	50+
B10305	White	HR	No	No	No	No	10+ yrs	50+
B10219	White	HR	No	No	No	No	<10 yrs	50+

nci, National Cancer Institute classification; HR, high-risk; SR, standard-risk; bcr, BCR-ABL1 translocation; e2a, E2A-PBX1 translocation; tel, Tel-AML translocation (*ETV6-RUNX1*).

Our results suggest that expression levels of *BCAR3*, *IL1R1*, *KCNH2*, *PIR*, and *ZDHHC23* make up the gene signature associated with high WBC count BP-ALL. The roles these genes play in the regulation of WBCs in the peripheral blood circulation and/or their association with prognosis remain to be determined. Pirin is an iron-dependent redox coregulator of NF-KB [5]. In acute myeloid leukemia, downregulation of Pirin contributes to myeloid differentiation arrest [6]. KCNH2 (hERG1; Kv11.1) is expressed in multiple cancers and controls cell proliferation and apoptosis [7, 8]. BCAR3 has been shown to regulate breast cancer cell migration, proliferation, and adhesion [9]. IL1R1 is the receptor protein for IL1α and IL1β, which are involved in B cell proliferation [10]. ZDHHC23 is involved in regulating expression of calcium-activated potassium channels [11].

We investigated protein-protein interactions and functional associations between the five proteins using STRING (PMID 25352553). The analysis revealed that there are no known direct interactions between the five proteins. To investigate indirect interactions between the five proteins we also considered the top 30 additional proteins from the STRING database having primary and secondary interactions with the five target proteins. The overall interactions between this set of 35 proteins (nodes) forms a connected graph with all nodes reachable, including the five target proteins. This result suggests that the five proteins may have common functional associations. Gene ontology analysis of the 35 proteins revealed that this set is significantly enriched for proteins involved in “immune response-activating signal transduction” (FDR *P* = 1.1e-^13^). Further studies to characterize the impact of dysregulation of these five genes on immune response signaling are warranted.

In conclusion, we identified, for the first time, genes linked to the peripheral WBC count in BP-ALL; *BCAR3*, *IL1R1*, *KCNH2*, *PIR*, and *ZDHHC23* expression make up the gene signature of high WBC count BP-ALL. Statistical analysis demonstrated that expression levels of the genes identified in this study could accurately categorize high and low WBC subjects using two independent patient sets, representing positive and negative *ETV6-RUNX1* cases. Our results, although in a small sample set, suggest that these genes may be involved in the underlying cellular mechanism leading to differences in WBC and patient prognosis and warrants further investigation in a prospective study. It would be interesting to determine if expression of these genes correlate with clinical outcome and establish them as independent prognostic markers. However, due to the patients being treated on various clinical trials over the past 15 years, the relationship with clinical outcome is not available. Further studies are in progress using leukemia cell line models to identify the role of these genes either individually or in combination in regulating WBCs in the peripheral circulation, which may aid in the development of prognostic factors or identify cellular pathways to target with new therapies.
